# Spermatozoon ultrastructure of hangingflies, *Bittacus strigosus* and *Bittacus stigmaterus*

**DOI:** 10.1093/jis/14.1.10

**Published:** 2014-01-01

**Authors:** Sally P. Shepardson, Brock A. Humphries, Kathleen L. Pelkki, David J. Stanton

**Affiliations:** Biology Department, Saginaw Valley State University, University Center, MI 48710

**Keywords:** electron microscopy, insect phylogeny, sperm

## Abstract

In the present study, spermatozoon ultrastructure was documented in two species of hangingflies, *Bittacus strigosus* Hagen (Mecoptera: Bittacidae) and B. *stigmaterus* Say. Structures considered important to phylogenetic assessment that were observed in *B. strigosus* and *B. stigmaterus* included a short bilayered acrosome, elongated nucleus, tube-like glycocalyx, centriole adjunct material, accessory bodies, two mitochondrial derivatives, extra axonemal rods, globular units, and 9+2 arrangement of microtubules in the axoneme. Comparisons were made to *Bittacus planus* Cheng, which was previously examined by electron microscopy ( Xie and Hua 2010 ). Similarities among the ultrastructural characteristics of the three *Bittacus* species support the monophyly of this genus. Displacement of a mitochondrial derivative by an accessory body was documented for the first time. This paper includes clarifications on differences between accessory bodies and extra axonemal rods, which are issues important to phylogenetic placement.

## Introduction

Comparisons of spermatozoon ultrastructure are often used to help establish phylogenetic relationships among insects ( Baccetti et al. 1969 ; Gassner et al. 1972 ; Jamieson et al. 1999 ; Dallai et al. 2003 ; Xie and Hua 2010 ). Documentation of the spermatozoon ultrastructure from additional members of the genus *Bittacus* has particular merit, since phylogenetic evaluation carries more validity with a greater number of described species. Therefore, the main purpose of this study was to record the spermatozoon ultrastructure of two species of hangingflies, *Bittacus strigosus* Hagen (Mecoptera: Bittacidae) and *B. stigmaterus* Say. The spermatozoon ultrastructural components of these species were also compared to *Bittacus planus* Cheng ( Xie and Hua 2010 ) to discover if the structures considered important for phylogenetic placement were present in all three species. These structures were a short bilayered acrosome, tube-like glycocalyx, extra axonemal rods (EARs), elongated nucleus, centriole adjunct material (CAM), two mitochondrial derivatives (MDs), accessory bodies (AB), 9+2 arrangement of axoneme microtubules, and globular units ( Dallai et al. 2003 ; Xie and Hua 2010 ).

Several previous studies have included ABs and EARs in their descriptions of spermatozoon ultrastructure ( Breland et al. 1966 ; Gassner et al. 1972 ; Dallai et al. 2003 ; Xie and Hua 2010 ), but identification of these structures has been inconsistent and confusing among the publications. Clarification of the differences between ABs and EARs was included in this study due to the importance of these structures in terms of phylogenetic considerations. Additionally, displacement of an MD by an AB was documented for the first time.

## Materials and Methods

Male members of *B. strigosus* and *B. stigmaterus* were collected in the Shiawassee National Wildlife Refuge near Saginaw, MI, in 2008 and 2009. All protocols complied with appropriate animal care committee poli-cies at Saginaw Valley State University and adhered to the legal requirements established in the United States regarding animal use and care in research. The testes were removed and fixed first in 3.0% glutaraldehyde in 0.1 M phosphate buffer, pH 7.2 with 1.8% sucrose, and kept for 24 hr to several months at 4°C. Post fixation occurred in the same buffer at 4°C and lasted 1 hr in 1.0% OsO4 at 4°C. Four 15 min buffer washes occurred after each fixation step. Samples were dehydrated at room temperature through a graded series of acetone at 15 min intervals and embedded in Spurr’s resin (Electron Microscopy Sciences, www.emsdiasum.com ). Light microscopy sections were cut at 0.5 µm, stained with 1.0% toluidine blue, and viewed with a Nikon Optiphot (Nikon Instruments, nikoninstru-ments.com). Micrographs were captured with Image Pro 5.1.2 software (Media Cybernetics, mediacy.com). For ultrastructure, sections were cut at 70 nm, stained with uranyl acetate and lead citrate, and viewed with a JEOL 1400 transmission electron microscope (JEOL, jeolusa.com) at 80 kV. Line drawings were created with Word Perfect Presentations (Corel, corel.com).

## Results

**Figure 1.**
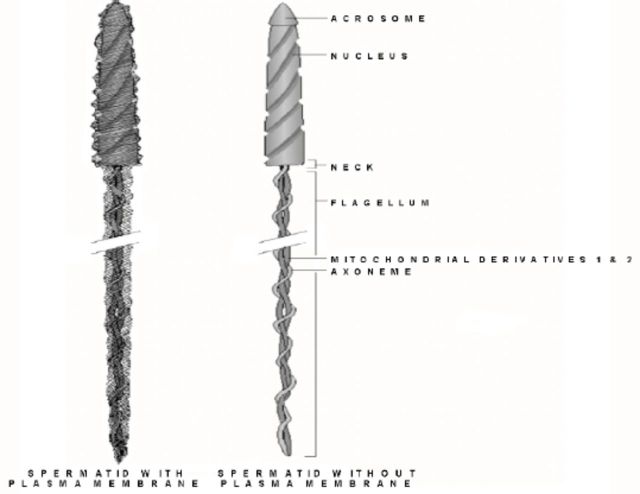
Line drawing of proposed spermatid general structure with and without the plasma membrane. Note the presence of both mitochondrial derivatives in the neck region. High quality figures are available online.

**Figure 2.**
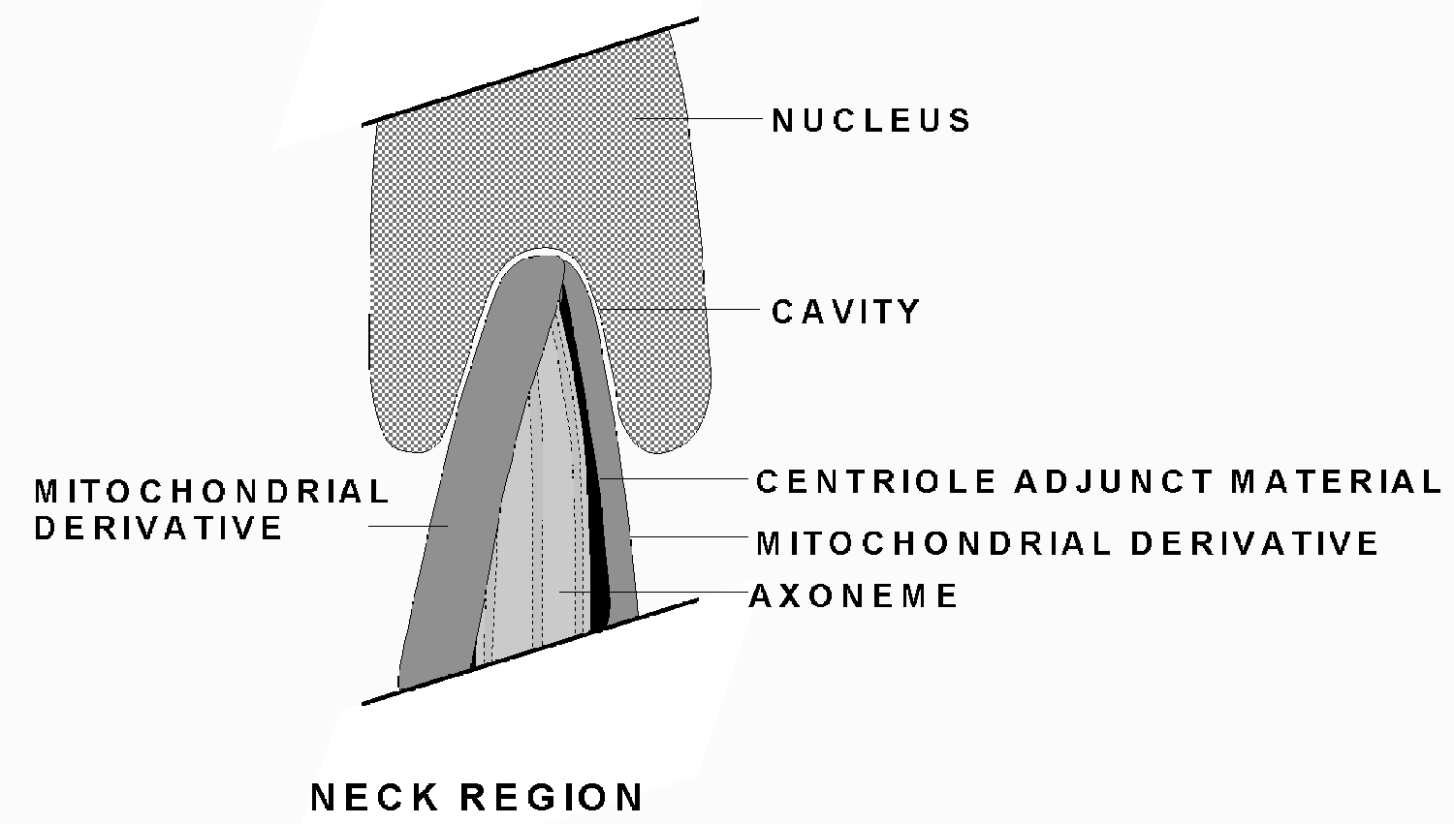
Line drawing of sectioned view of the proposed spermatid neck region. Note the presence of both mitochondrial derivatives as well as the axoneme and partial centriole adjunct material. High quality figures are available online.

**Figure 3.**
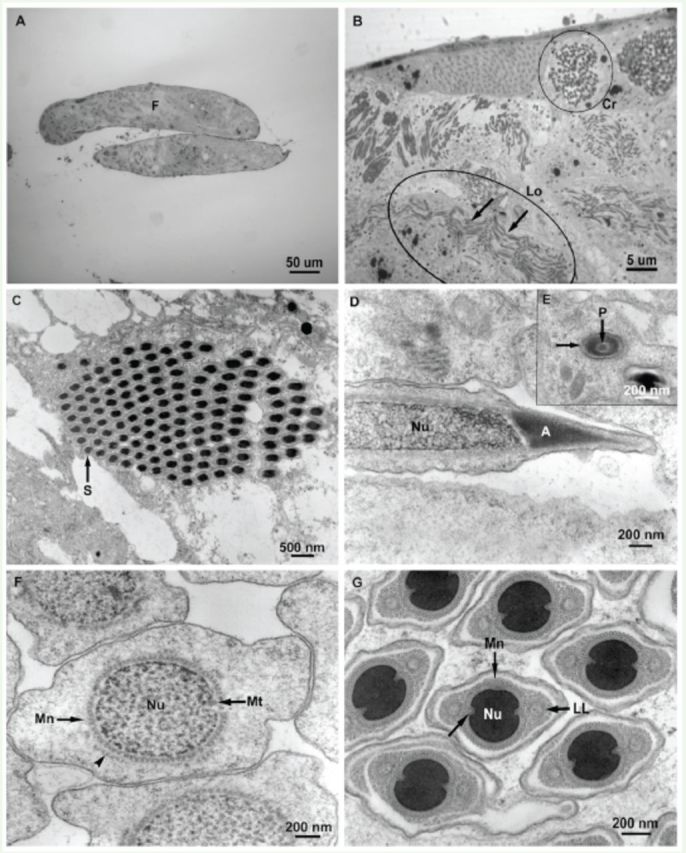
A, D–G. *Bittacus strigosus.* C corresponds to *B. stigmaterus* . A. Light microscopy, longitudinal section of typical banana shaped follicles (F) of the testis. B. Light microscopy, higher magnification of A. C–G. Electron microscopy, cysts showing cross sectional (Cr) and longitudinal (Lo) orientation of the spermatozoa; undulation of spermatozoa (arrows) C. Cross section of cyst containing 128 spermatozoa (S). D. Longitudinal section of acrosome (A) of spermatid nucleus (Nu); note uncondensed nuclear material. E. Cross-section of acrosome with outer conical acrosomal vesicle (arrow) surrounding the perforatorium (P). F. Spermatid nucleus (Nu) surrounded by the manchette (Mn); note that the manchette is double layered in some locations (arrowhead); microtubule in indentation (Mt). G. More mature spermatid nucleus (Nu) with condensed DNA, nuclear grooves (arrow), lateral lamellae (LL), and single-layered manchette (Mn). High quality figures are available online.

Since spermatozoon ultrastructure of *B. strigosus* and *B. stigmaterus* was so similar, only representative figures from *B. strigosus* are presented, with the exception of Figure 3C , which is from *B. stigmaterus* . Structures of spermatids and spermatozoa were emphasized. Line drawings made from compilations of electron micrographs from both *B. strigosus* and *B. stigmaterus* , and representing the generalized spermatid ultrastructure from these two species, are in Figures 1 and 2 .

### Follicles and cysts

Light microscopy micrographs showed the typical banana-shaped follicles of the testis ( Figure 3A ). Higher magnifications revealed that the spermatozoa-containing cysts within the follicles were aligned at various angles throughout the follicle, providing cross, longitudinal, and tangential views of the spermatozoa regardless of the orientation of the follicle when sectioned ( Figure 3B ). Follicle cysts contained 128 mature spermatozoa ( Figure 3C ), indicating seven divisions from the spermatogonial cell. It was apparent that the spermatozoa undulated in the sections ( Figure 3B ), and no single view of an entire spermatozoon was possible in thick or thin sections.

### Acrosome, nucleus, plasma membrane, and glycocalyx

**Figure 4.**
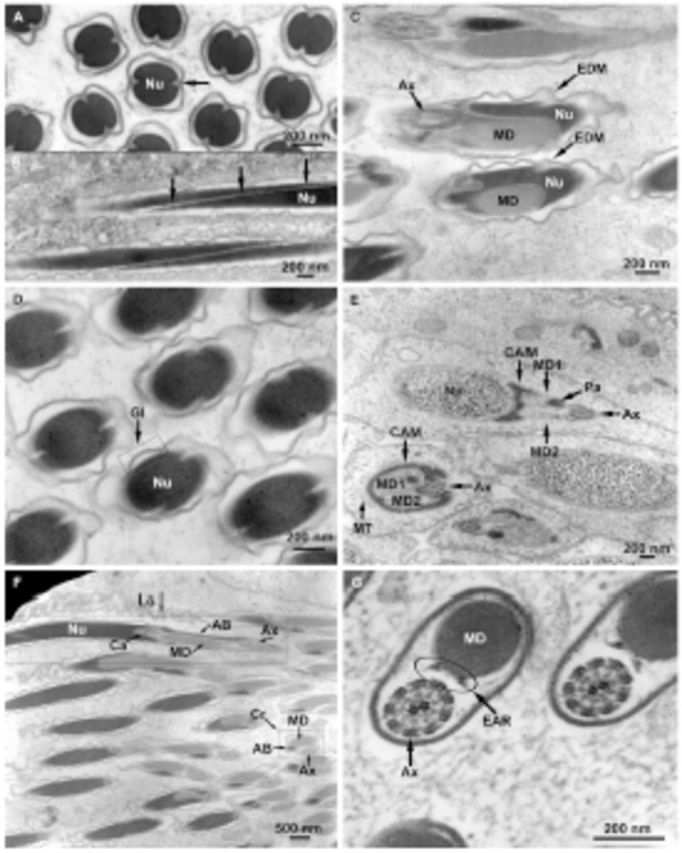
*Bittacus strigosus* , electron microscopy. A. Cross section of spermatozoa showing loss of most cytoplasm; mature nucleus (Nu); nuclear grooves (arrow). B. Longitudinal section of spermatozoa showing loss of most cytoplasm; mature nucleus (Nu) revealing the nuclear grooves (arrows) along the entire nucleus. C. Electron dense material located at the grooves of the nucleus (Nu); mitochondrial derivative (MD); axoneme (Ax). D. Tangential view of the mature nucleus (Nu) showing the tubular glycocalyx (Gl). E. Centriole adjunct material (CAM) extending around the mitochondrial derivatives (MD1, MD2) in a partial sheath of a spermatid, note the uncondensed nuclear material of the nucleus (Nu) and the presence of both mitochondrial derivatives in the neck region; axoneme (Ax); paracrystalline material (Pa); microtubules (MT). F. Neck region of spermatozoa showing longitudinal (Lo) and cross sections (Cr), note only one mitochondrial derivative (MD) is present; nucleus (Nu); accessory body (AB); axoneme (Ax); nuclear cavity (Ca). G. The 9+2 arrangement of the axoneme (Ax); extra-axonemal rods (EAR); presence of single mitochondrial derivative (MD) indicates the posterior end of the flagellum. High quality figures are available online.

**Figure 5.**
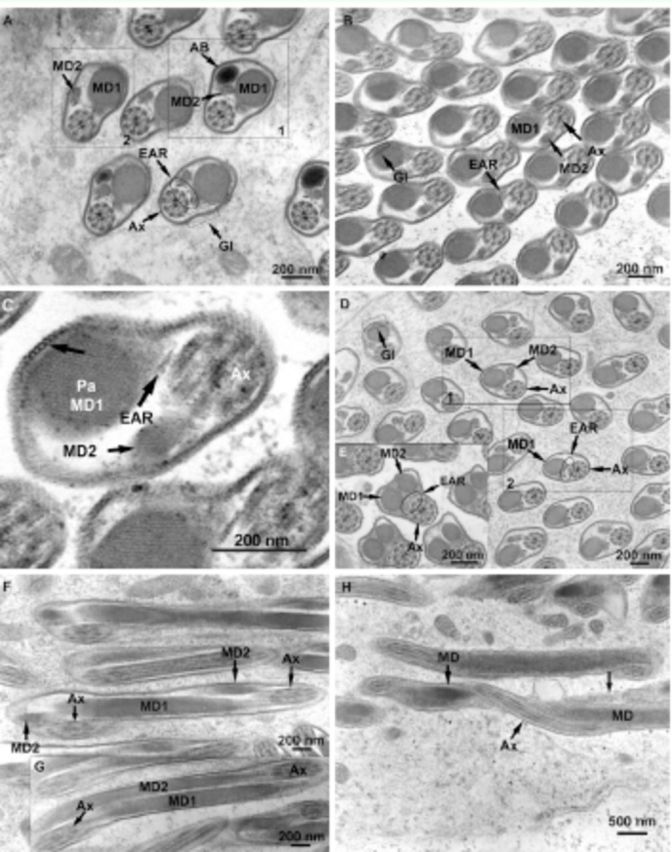
*Bittacus strigosus* , electron microscopy. A. Transitional area showing the disappearance of the accessory body (AB) near the original mitochondrial derivative (MD1), and the appearance of a second mitochondrial derivative (MD2) (Boxes 1 and 2); extra axonemal rods (EAR); axoneme (Ax); tubular glycocalyx (Gl). B. Area more posterior along the flagellum, compared to Figure 5A where the accessory body has completely disappeared and the second mitochondrial derivative (MD2) has taken its place; extra axonemal rods (EAR); tubular glycocalyx (Gl); axoneme (Ax); mitochondrial derivative (MD1). C. Tangential section of flagellum near its posterior end showing the tubular glycocalyx (arrow); mitochondrial derivatives (MD1, MD2); cross-hatched paracrystalline material (Pa) in MD1; axoneme (Ax); only one extra axonemal rod (EAR) is distinct due to the angle of the section. D. Further along the flagellum from Figure 5E , one mitochondrial derivative (MD2) begins to taper (Box 1) and eventually ends (Box 2); extra axonemal rods (EAR) are still present near the axoneme (Ax); tubular glycocalyx (Gl). E. The mitochondrial derivatives (MD1, MD2) develop a similar diameter for some of the flagellum length after MD2 replaces the accessory body; extra axonemal rods (EAR); axoneme (Ax). F. At some locations in the flagellum, the mitochondrial derivatives (MD1, MD2) wrap around each other; note that the axoneme (Ax) wraps around both mitochondrial derivatives. G. The two mitochondrial derivatives (MD1, MD2) run parallel at other places in the flagellum; note that the axoneme (Ax) wraps around both mitochondrial derivatives. H. The axoneme (Ax) wraps around the single mitochondrial derivative (MD) near the end of the flagellum; note the globular units (arrow). High quality figures are available online.

The acrosome was of the short bilayer type; the outer conical acrosomal vesicle surrounded an inner rod, the perforatorium ( Figures 3D , E ). The nucleus elongated as the spermatids matured, and a sheath of microtubules, known as a manchette and composed of one full and one partial layer of microtubules, surrounded the nucleus prior to DNA condensation. Two indentations at opposite sides of the nucleus, each containing one microtubule, were present at this stage ( Figure 3F ). Later in spermatid maturation, as the DNA condensed, two lateral lamellae formed near the indentations ( Figure 3G ). The lateral lamellae were bounded by a double membrane surrounded by microtubules and frequently contained one or two microtubules. The manchette was then mostly single-layered, and the nucleus developed grooves at the site of the indentations ( Figure 3G ). The spermatozoon lost the majority of its cytoplasm, manchette, and lateral lamellae ( Figures 4A , B ), and a cavity developed at the posterior end of the nucleus ( Figures 2 , 4F ). In longitudinal sections, it was evident that grooves spiraled along the long axis of the nucleus ( Figure 4B ), apparently matching a twisting of the entire nucleus ( Figure 1 , morphological reconstruction). The plasma membrane was loosely opposed to the nucleus, but electron-dense material existed in the nuclear groove areas ( Figure 4C ), implying attachment points between the plasma membrane and the nucleus. In tangential sections, a glycocalyx in a tu-bule-like formation was apparent ( Figure 4D ). Because the cross and longitudinal sections of these tubules did not occur in true cross and longitudinal sections of the nucleus, it was concluded that the tubules were arranged at an angle relative to the longitudinal axis of the spermatozoon. The tubules decorated the spermatozoon throughout its entire length ( Figures 4D , 5A–D ).

### Centriole adjunct material, mitochondrial derivatives, accessory body, and axoneme

As the spermatids matured, the CAM occupied an incomplete cylinder in the neck region around the axoneme and two MDs that were present in the neck region early in the spermatid maturation process ( Figure 4E ). Extension of the CAM, termed an AB ( Dallai et al. 2003 ), continued some distance down the flagellum ( Figure 4F ). Beginning at the neck region, the axoneme was wound around the two (early in spermatid maturation) or one (spermatozoon) MDs (see next section and discussion) along the entire length of the flagellum ( Figures 1 , 4E , 5F–H ). This was apparent because the axoneme appeared in a cross or tangential section relative to the MDs in those figures. The cytoskeletal elements within the axoneme were in the 9+2 arrangement, and there were no accessory tubules ( Figure 4G ).

### Neck region, flagellum, and extra axonemal rods

The spermatozoon neck region was where the nucleus met the flagellum ( Figures 2 , 4E–F ). In the spermatid, recognizable by the lack of fully condensed DNA and a limited posterior nuclear cavity ( Figure 4E ), the initial location of the two MDs and axoneme was close to the base of the nucleus ( Figures 2 , 4E ). One MD was larger in diameter than the other, and the larger MD appeared to begin the paracrystalli-zation process before the smaller MD ( Figure 4E ). Microtubules were observed to be closely opposed to the MDs ( Figure 4E ). Only one MD, the axoneme, and the AB were present in the posterior nuclear cavity of the spermatozoon, which was recognizable by the condensed nuclear material and distinct posterior nuclear cavity ( Figure 4F ). Sections that included both longitudinal and cross sections were studied ( Figure 4F ) due to concerns that sections were too thin to reveal a possible second MD if the two MDs lay side by side in a longitudinal view and the plane of the section sliced through only one MD. It is apparent from the cross sections that there was only one MD present in the neck region of the spermatozoon ( Figure 4F ).

At a distance further down the flagellum, a transition area in the spermatozoon occurred in which the AB decreased in diameter and ended and the second MD appeared ( Figure 5A ). The diameters of the two MDs were sub-stantially different. The AB and the two MDs appeared together for some distance along the flagellum until the AB ended ( Figure 5A ). EARs, either round or somewhat triangular in diameter, were apparent for the first time in this same area and continued down the flagellum ( Figures 4G , 5A–E ). Further along the flagellum, the diameters of the two MDs became more similar ( Figure 5E ) until another transitional area was reached where one of the MDs eventually ended ( Figure 5D ; cross section near end of flagellum, Figure 4G ).

When present together, the two MDs either wound around each other along the flagellum ( Figure 5F ) or ran parallel to each other along the flagellum ( Figure 5G ). The axoneme wrapped around the outside of the MDs in both cases ( Figures 5F , G ). The axoneme also wrapped around the single MD ( Figure 5H ), which occurred at the posterior end of the flagellum. Globular units were present ( Figure 5H ). No deterioration of the axoneme at the end of the flagellum was apparent.

## Discussion

The spermatozoon ultrastructure of *B. stigmaterus* and *B. strigosus* was documented in this study, adding important information about these two species. The validity of phylogenetic analysis of *Bittacus* is increased by the availability of a larger number of species for ultrastructural comparison. In *B. strigosus* and *B. stigmaterus* , the finalized acrosome was of the short bilayer type with a prominent perforatorium. At the later stages of spermatid maturity in *B. strigosus* and *B. stigmaterus* , the nucleus underwent a dramatic elongation. The spermatozoon nucleus had a posterior cavity and a loosely-apposed plasma membrane, and grooves were noted. *Bittacus strigosus* and *B. stigmaterus* had a distinct tu-bule-like structure in the glycocalyx, and this membrane decoration was continuous along the entire length of the spermatozoon. Accessory tubules were absent in the axoneme, resulting in the 9+2 configuration of microtubules. These are all features also noted in *B. planus* ( Xie and Hua 2010 ).

At the anterior end of the spermatozoon flagellum in *B. strigosus* and *B. stigmaterus* , the CAM formed an incomplete sheath around the axoneme and MDs, and one part of the CAM extended further down the flagellum. This extension is known as an accessory body (AB) ( Dallai et al. 2003 ). In Mecoptera, there have been consistent reports of the presence of CAM and ABs in spermatozoa, but there is variation in its description among the species ( Baccetti et al. 1969 ; Gassner et al. 1972 ; Dallai et al. 2003; Xie and Hua 2010 ). In *B. planus* , two ABs that extended down the length of the flagellum were observed ( Xie and Hua 2010 ). As noted in the discussion of the EARs below, an alternate interpretation of the identification of AB vs. EAR, proposed by Xie and Hua (2010) , may be justified.

MDs in *B. planus* ( Xie and Hua 2010) , *B. strigosus* , and *B. stigmaterus* had similar configurations. Initially, there were MDs of different diameters in the neck region, then MDs of the same diameter further along the flagellum. Finally, one MD terminated before the end of the flagellum. This study of *B. strigosus* and *B. stigmaterus* presents the first evidence of an AB displacing one of the two MDs originally located at the extreme anterior end of the flagellum at the base of the nuclear cavity in the spermatid flagellum. In the spermatozoon flagellum, only one MD was observed at the base of the nuclear cavity with the then-extended AB. The AB eventually ta-pered off and ended at the same level of the flagellum where a second MD was again seen. The inference is that as the AB formed by extension of the CAM, one of the MDs was relocated from its original location in the neck region, near the base of the nucleus, to start at the place where the AB ended. There were microtubules present in immature flagella that could assist such a process. Perhaps a particular diameter of the flagellum must be maintained for proper movement, and the presence of both MDs, the axoneme, and the AB would violate that diameter in *B. strigosus* and *B. stigmaterus* . This new demonstration of a relocated MD may account for the variety of observations regarding position and length of the ABs noted above, since stage of maturation may be essential in determining the morphological appearance of this structure.

Both *B. strigosus* and *B. stigmaterus* contained EARs, structures first discovered in *Panorpa germanica* ( Dallai et al. 2003 ). Structures similar to the EARs were seen in *B. planus* (Figures 7D, E and 8A–C), wherein the structures were labeled as ABs ( Xie and Hua 2010 ). There appears to be some confusion in the literature between ABs and EARs (Baccetti et al. 1969; Gassner et al. 1972 ; Dallai et al. 2003 ; Xie and Hua 2010 ), but there are morphological differences separating ABs and EARs that can be used to distinguish between them. The ABs typically remain less distinct in outline, whereas the EARs often develop sharper edges. Additionally, the EARs tend to be more triangular in shape. Using these criteria, it is proposed that the structures noted in the *Bittacus* genera discussed in this para-graph may be various stages of EAR development. According to this hypothesis, the EARs are originally more fibrous in nature and eventually taper down to smaller diameters until, in some cases, they resemble trian-triangles. It is also possible that the different appearance of the presumed EARs in the various species is another example of the variation in characters already found in Mecoptera, such as the length of ABs and MDs.

Previous studies have suggested that the presence of the following spermatozoon characters are important to phylogenetic placement of insect species in Mecoptera, specifically *Bittacus* : EARs, tubular glycocalyx, elongated nucleus, CAM, ABs, two MDs, 9+2 arrangement of microtubules in the axoneme (no accessory tubules), globular units, and short, bilayered acrosome ( Dallai et al. 2003 ; Xie and Hua 2010 ). This study documented all of these characters in *B. strigosus* and *B. stigmaterus* and they have also been found in *B. planus* ( Xie and Hua 2010 ). Table 1 summarizes this information and contains the dog flea, *Ctenocephalides canis* Curtis (Siphonaptera: Pulicidae), ( Dallai et al. 2003 ) for comparison. Taken in this context, the spermatozoon ultrastructural characters of *B. strigosus* and *B. stigmaterus* documented in this study help establish the concept of monophyly in *Bittacus.* No conflicting spermatozoon ultrastructure in *B. strigosus* and *B. stigmaterus* that can be used as evidence of paraphyly was found. More studies that include many more species are necessary to conclusively determine monophyly within *Bittacus* .

**Table 1.**
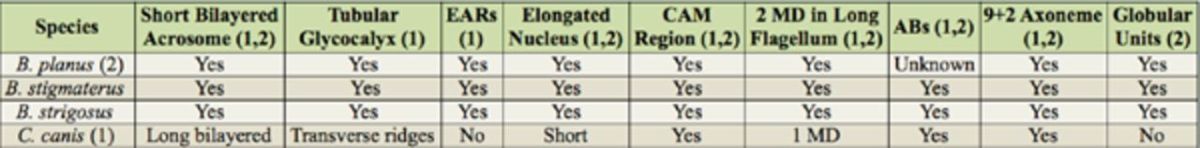
Comparison of spermatozoon ultrastructural characters among three *Bittacus* species and one Siphonapteran ( *Ctenocephalides canus* ).

1= Dallai et al. 2003 ; 2= Xie and Hua 2010 “Unknown” means that information on the character was ambiguous in the published study. Numbers by the characters indicate that the character was selected as significant for phylogenetic analysis by the authors of the relevant paper. The number by *Bittacus planus* and *Ctenocephalides canus* indicates the study that produced the spermatozoon ultrastructural information listed in Table 1 .

## Abbreviations

AB: accessory body
CAM: centriole adjunct material
EAR: extra axonemal rod
MD: mitochondrial derivative
